# Applications of Microsatellite Markers for the Characterization of Olive Genetic Resources of Tunisia

**DOI:** 10.3390/genes12020286

**Published:** 2021-02-18

**Authors:** Olfa Saddoud Debbabi, Sameh Rahmani Mnasri, Fathi Ben Amar, M’barek Ben Naceur, Cinzia Montemurro, Monica Marilena Miazzi

**Affiliations:** 1 Banque Nationale de Gènes, Boulevard du Leader Yesser Arafet, Charguia 1, 1080 Tunis, Tunisia; mnasrisameh@yahoo.fr (S.R.M.); dg_bng@bng.nat.tn (M.B.N.); 2 Institut de l’Olivier, University of Sfax, Route de l’Aeroport Km 0.5, 3029 Sfax, Tunisia; fathibenamar@yahoo.fr; 3 Department of Soil, Plant and Food Sciences (DISPA), University of Bari, Via Amendola 165/A, 70126 Bari, Italy; monicamarilena.miazzi@uniba.it

**Keywords:** fingerprinting, genetic diversity, olive, plant genetic resources, SSR

## Abstract

Among the countries of the Mediterranean Basin, Tunisia is located at the crossroad for the immigration of several civilizations over the last two millennia, becoming a strategic place for gene flow, and a secondary center of diversity for olive species. Olive is one of the principal crop species in Tunisia and now it strongly characterizes the rural landscape of the country. In recent years, collecting missions on farm and *in situ* were carried out by various institutes, with special emphasis given to *ex situ* collections serving as a reference for the identification of olive germplasm. Simple Sequence Repeats (SSRs) represent the easiest and cheapest markers for olive genetic fingerprinting and have been the tool of choice for studying the genetic diversity of this crop in Tunisia, to resolve cases of homonymy and synonymy among the commercialized varieties, to identify rare cultivars, to improve knowledge about the genetic variability of this crop, to identify a hot spot of olive biodiversity in the Tunisian oasis of Degache, and to enrich the national reference collection of olive varieties. The present review describes the state of the art of the genetic characterization of the Tunisian olive germplasm and illustrate the progress obtained through the SSR markers, in individuating interesting genotypes that could be used for facing incoming problems determined by climate changes.

## 1. Introduction

Tunisia was at the crossroads different civilizations for several millennia becoming a reservoir for gene exchange and flow of genetic resources for numerous Mediterranean crops for which it shows a large genetic variation including in olive (*Olea europaea* L.). The most iconic tree of the Mediterranean Basin has origins linked to the most ancient civilizations, about six millennia ago [1,2,3]. Today, with more paleobotanical, archaeological, historical and molecular data available, it is easier to trace the history of the olive tree species [4].

In Tunisia, the history of the olive dates back to Phoenicians and, later, Romans, whose intense trades contributed to expand the cultivation areas, promoting the diversification of the olive germplasm through the genetic flows and introgression of alleles from other subspecies of *O. europaea* [5,6]. Tunisia is considered a secondary center of diversification for the olive crop, which has been a pillar of the Mediterranean diet since the most ancient times. The coexistence of both wild and cultivated forms in the Mediterranean vegetation poses challenges to researchers in understanding the domestication history of this species. Plant domestication is considered a major factor that attends the evolution of genomes, while adaptation generally occurs after domestication and it is associated with phenotypic changes [7].

Cultivated olive (2n = 2x = 46; genome size 1800 Mb) is an evergreen, high longevity, predominantly allogamous, vegetatively propagated tree [8]. About 95% of the world’s olives are produced in the Mediterranean Basin. At international level, within the 47 olive growing countries, more than 3,300,000 tons of olive oil are annually produced [9] on an area of over 10.8 million ha, ranking 7th among all vegetable oils produced worldwide, and 25th among the 160 cultivated crops in the world [10]. In Tunisia, olive cultivation holds 82 million olive trees covering an area of 1.84 million hectares [11] localized 30% in the north, 38% in the centre and 32% in the south [12]. It represents 40% of the overall value of agronomic exports of the country and it gives Tunisia the rank of fourth largest producer, and third largest exporter of olives and olive products in the world [13].

**Figure 1:**
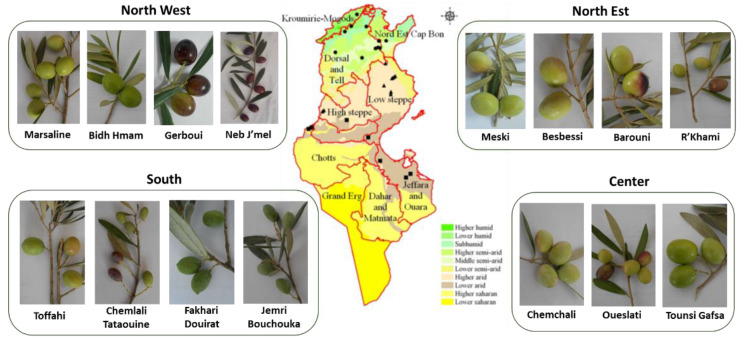
Diversity of some olive varieties cultivated in different areas of Tunisia (Photograhs of F. Ben Amar, Olive Tree Institute).

Although the olive germplasm in Tunisia is represented by more than 200 cultivars and genotypes [14] (Figure 1), only few cultivars are largely cultivated.

**Figure 2:**
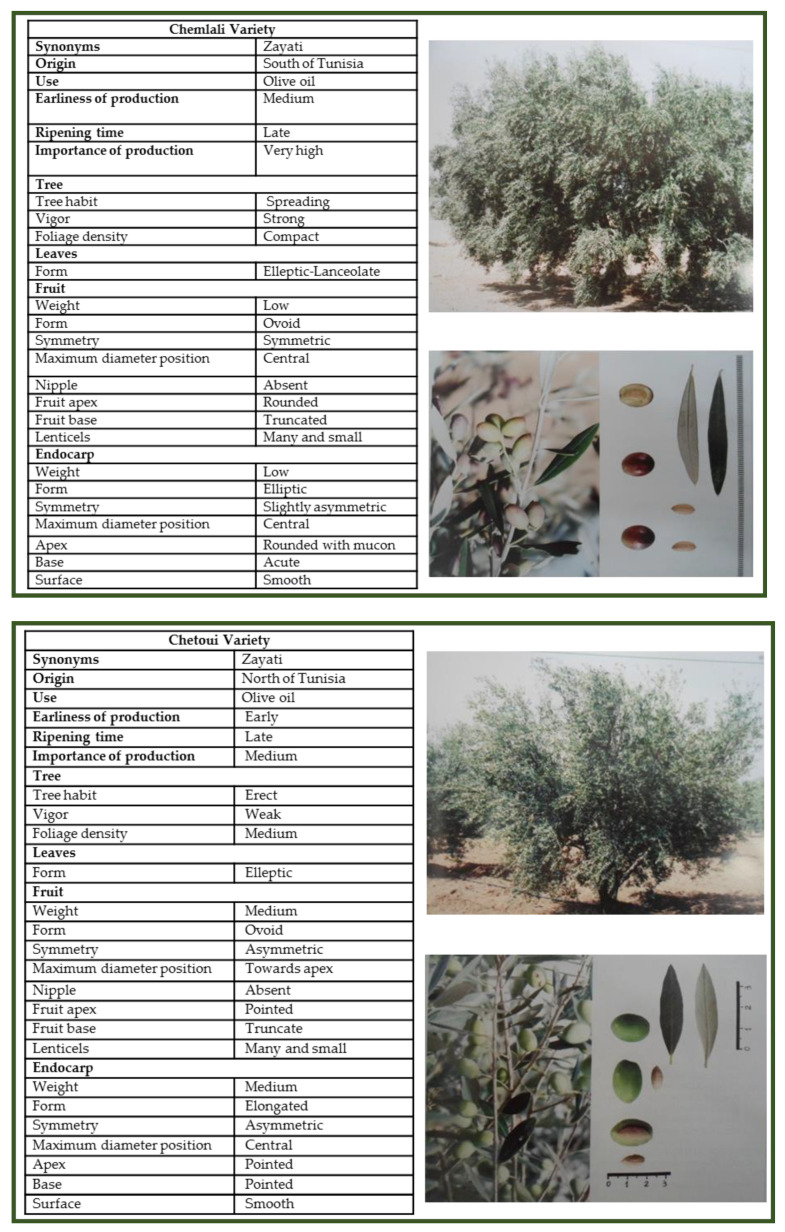
Description and passport data of most cultivated Tunisian varieties Chetoui and Chemlali, as described by Trigui and Msallem [15].

The production of Tunisian olives is almost dominated by only two varieties, “Chetoui” in the north, and “Chemleli” in the center and south of the country (Figure 2), while several minor varieties are still present in restricted areas. 

In addition to the 56 cultivars identified by Trigui and Msallem [15], many “ecotypes” are grown in rural areas where traditional cultivation systems prevail. These varieties are adapted to specific geographical conditions and agricultural practices in various agricultural systems and are preserved by farmers as a personal heritage.

A growing awareness about the importance of preserving genetic resources from erosion as source of genes of agronomic interest, has prompted government authorities in several countries, to focus on saving agrobiodiversity. Additionally, programs have been implemented to help farmers manage and conserve their plant genetic resources which are crucial to food security and sustainable agriculture for present and future generations [16]. Tunisia was among the first signatories of the International Treaty on Plant Genetic Resources for Food and Agriculture (ITPGRFA) and the Nagoya Protocol in 2011. The National Genebank of Tunisia is adopting both *ex situ* and on farm conservation strategies to sustainable management of local agrobiodiversity and use. The conservation of the genetic resources in the area where they have evolved (on farm conservation) is integrating strategies based on genebanks (*ex situ* conservation), especially for wild relatives of cultivated plants. When on farm conservation is adopted, the genetic diversity of cultivated native varieties is preserved within traditional farming systems. This diversity is guarded by custodian farmers who safeguard genetic resources for their families and communities. In general, smallholder farmers contribute to the conservation of genetic diversity by maintaining traditional varieties and minor genotypes adapted to local conditions. 

The development of the concept of *ex situ* conservation has made it possible to improve the techniques and means used to preserve genetic resources. *Ex situ* conservation corresponds to the conservation of the biological components of diversity outside their natural habitats [17]. This conservation strategy requires sampling, transfer and storage of collected accessions in genebanks in appropriate conditions to maintain their viability. The most important collections have been established and preserved for the global community by the Consultative Group for International Agricultural Research (CGIAR), for over 40 years. In the agreements signed in 1994 by the CGIAR centers with Food and Agriculture Organisation (FAO), their collections were integrated into the international network of the *ex situ* collections of the International Treaty on Plant Genetic Resources for Agriculture and Food. According to the World Information and Early Warning System on Plant Genetic Resources (WIEWS), it is estimated that around 7.5 million entries are actually preserved worldwide with around 6.6 million held in national government genebanks, the 45% of which is in just seven countries, with a downward trend if compared to the 12 countries reported in 1996 [10].

Genomic DNA-based markers have enormously empowered our ability to characterize genetic variation in crop plants, in particular simple sequence repeats (SSRs) or microsatellites. SSRs are hypervariable short (1–6 bp) repeat motifs that show a high level of length polymorphism due to insertion or deletion mutations of one or more repeat types, highly distributed throughout the genome [18]. Introduced in plant genetics in the early 90s, since then, their use increased due to their efficiency coupled with time saving [19]. Microsatellites are co-dominant, highly polymorphic, reproducible, and they are not affected by plant tissue types, developmental stages, and environmental factors [20].

In olive, microsatellite regions were sequenced only in the early 2000s [21,22]. They were firstly used to unravel the origin of the species [23] and its relationships with the wild olive [24]. Since then, they have been used to address many aspects of *O. europaea* L. genetics, such germplasm diversity [25,26,27,28], phylogenetic studies [29,30], paternity analysis [31,32,33], construction of linkage maps [34,35]. All their advantages over other markers made the use of standardized sets of SSR markers as a routine for variety fingerprinting [36,37,38,39] and promoted the development of new Expressed Sequence Tags (EST)-SSRs. By *de novo* next generation transcriptome sequencing of *O. europaea*, Dervishi et al. 2018 [40] identified eight highly informative EST-SSRs. Transcriptome libraries developed from different tissues of several olive varieties allowed Mariotti et al. [41] to select 26 new SSRs with high discrimination power within the *Olea* taxon, also highlighting their potential application as functional markers. The increasing economic value of the olive tree has increased the use of SSRs also for oil traceability and authenticity, sometimes in combination with Single Nucleotide Polymorphism (SNP) markers [42,43]. 

Despite the high use of throughput omics data for olive species, very few studies describing the state of art exist. This paper will focus on the contribution of molecular markers, particularly SSRs, in their utility and their use for a better exploration of Tunisian olive germplasm.

## 2. Management of *Ex Situ* Collections Using SSR Markers

So far, we number more than 100 collections of olive genetic resources at international, regional and national levels, with an increasing expansion of their number. The International Olive Council, with the aim to protect olive patrimony, created a network of 23 olive germplasm banks, housing over 1700 varieties. This network is composed of 3 international banks—Cordoba (Spain), Marrakech (Morocco) and Izmir (Turkey)—and 20 national banks, including one in Tunisia [44,45,46]. SSRs have been the markers of choice for the evaluation of these olive collections.

The Worldwide Olive Germplasm Bank of Córdoba (WOGBC) (Spain) conserves 900 accessions from 25 countries that were characterized through morphological and SSR markers [44]. It includes 361 Mediterranean olive accessions which were classified in 3 gene pools based on both country origin and genetic structure, i.e., East Mediterranean (mostly from Cyprus, Egypt, Lebanon, and Syria), West Mediterranean (mostly from Morocco, Spain, and Portugal), and Central Mediterranean (mostly from Algeria, Italy, Slovenia, Croatia, Tunisia, and Greece) [46].

To guide conservation decisions for an effective protection of the endangered species as sources of valuable alleles for the future needs, optimizing the conservation of a species while reducing the cost, it is crucial to establish reduced collections which represents, in limited size, the genetic diversity of the crop with the minimum similarity between its entries [47,48]. To increase the efficiency of characterization and utilization of collections stored in the genebanks, while preserving as much as possible the genetic diversity of the entire collection, the concept of core collections was introduced [49]. However, this can be achieved only through assessing the amount and distribution of genetic diversity in populations from different locations.

From the whole collection of the WOGBC, using agronomical traits and molecular markers (SSR, SNP, Diversity Arrays Technology (DArT)), a core collection was established, composed of 68 accessions suitable for genetic conservation, and including 36 accessions with potential desirable traits for olive breeding [50]. Gómez-Rodríguez et al. [51]. proved that 4 selected SSR markers were enough to distinguish the 36 cultivars of this core collection at WOGBC. 

SSR markers were used to establish core collections from the world Olive Germplasm Bank of Marrakech, that encompasses the whole Mediterranean genetic diversity, holding 561 accessions from 14 Mediterranean countries [52], as well as from the Worldwide Olive Germplasm Bank of Izmir (Turkey) which holds 500 accessions from 17 countries [53]. Many others olive minor collections were implemented and characterized by SSR markers in several countries, such as Greece [54], France [55,56,57], Algeria [58], United States [59], Italy [60,61,62], Portugal [63,64] and Albania [65].

In Tunisia, several *ex situ* olive collections have been established, mainly by the Olive Tree Institute. The plant material has been characterized mainly by morphological and biochemical markers [15,66], but the dependence of these markers on environmental conditions makes them limited and obsolete compared to molecular markers such as Random Amplified Polymorphic DNA (RAPD), Amplified Fragment Length Polymorphism (AFLP), or, more recently, SSR and SNPs which have become, today, the markers of choice for olive genetic characterization [67,68,69,70].

The Boughrara Collection, implemented at the Olive Tree Institute, is considered the National collection in Tunisia, and it holds more than 201 varieties, 147 of which are indigenous (73.13%) [71].

For their characterization, eight SSR markers and endocarp morphological characters were initially used in a study by Fendri et al. [72] which led the identification of 84 accessions, resolving cases of homonymy and synonymy. Complementarily, Saddoud Debbabi et al. [14], using the 12 SSRs (Table 1) most used at international level [21,59], genotyped 26 cultivars from the olive national collection, representing the main varieties cultivated in Tunisia (Table 2).

These genotypes are conserved at the national field of the National Gene Bank of Tunisia (NGBT) and they serve as a safety duplicate collection. This work allowed also the implementation of a national database of SSR data, and the genotyping of the 12 main commercialized cultivars to be proposed for the national varietal certification of Tunisian olive germplasm, and to be used to guarantee the genetic authenticity of commercial varieties [14].

## 3. Olive Genetic Resources Characterization and Study of Genetic Diversity

Due to the growing economic importance of the olive tree, studies aimed at characterizing the main varieties marketed have multiplied. In the meantime, in the light of the impact of climate change on crop performance and productivity [73], the need to identify, preserve and exploit varieties adapted to difficult conditions, such as extreme variations in rainfall and temperatures, has become crucial. Thus, the characterization and conservation of old olive cultivars is also a priority, because they face a growing risk of extinction due to urbanization and the introduction of new commercial varieties [5].

Using SSRs markers, numerous works of olive genotyping were carried out in Tunisia, allowing the tracing of an overview of the results achieved on the conservation of olive genetic resources in this country.

Many researches focused on the main cultivated varieties. Taamalli et al. [74] investigated the diversity within the two major cultivars Chemlali and Chetoui, showing that the latter group does not have any significant relationship with any other cultivar, and it very likely to derive from a single clone, while Chemlali and Chemchali cultivars show some degree of heterogeneity. It was also pointed out a high degree of similarity between “Zalmati” and “Chemlali-Sfax”, which is commercially troubling and should be addressed in greater deeper detail using a larger number of markers.

SSR were applied to distinguish “Chemlali” samples originating from different parts of Tunisia, from the “Zarrazi” variety, typically cultivated in the south of Tunisia. The polyclonal cultivar “Chemlali” is a mixture of closely related genotypes, and it seems to provide evidence for the exchange of olive cultivars throughout different regions and of gene flow between genotypes [75]. Among Tunisian varieties, the cultivar “Chemlali” should be the most important target of conservation, based on its contribution to diversity, in particular in southern Tunisia where it represents an important reservoir of genetic diversity.

Considering the overall Tunisian olive germplasm, Taamalli et al. [75] observed the presence, of two main gene pools referring to the north and the south of the country, suggesting a possible relationship with the different climatic conditions in the two areas. On the contrary, focusing on the characterization of South East Tunisian germplasm, Ben Mohamed et al. [76], and Ben Ayed et al. [77] highlighted a lack of correlation between genetic and geographical origin of olive cultivars in Tunisia. Other authors [78] have shown the clustering of cultivars from the same or a nearby region, suggesting a common genetic base of these cultivars. This is in agreement with the hypothesis of autochthonous origin of most of the olive cultivars as well as their limited diffusion from their centers of origin [79]. In fact, cultivar intercrossing and crosses with wild accessions, along with local selection of outstanding seedlings and subsequent vegetative cloning, could have led to a large number of varieties around their possible original areas of cultivation.

The classification of Tunisian olive varieties based on SSR molecular markers is highly correlated with the form and the weight of the fruits and the endocarps, thus demonstrating the efficiency of using qualitative morphological markers to discriminate olive varieties [26]. Likewise, the same authors confirmed the absence of correlation between the molecular clustering of Tunisian varieties and their geographical distribution and proved that the gene flow of the species *Olea europaea* L. in Tunisia is highly influenced by the empirical selection achieved by spontaneous crossing and the exchange of propagation material among growing areas.

The richness of the Tunisian olive gene pools was demonstrated by Saddoud Debbabi [14] in a collection of 31 minor cultivars and 26 reference olive varieties characterized by using 12 microsatellites. The authors highlighted an overall high genetic diversity of the marginal germplasm, particularly for the cultivars collected from the regions of Ras Jbal and Azmour. The research pointed out, in this unknown germplasm, gene pools not present in commercial (Nurseries) varieties, underlining the need to broaden the genetic base of the commercialized germplasm to avoid genetic erosion of this olive patrimony. In addition, Saddoud Debbabi et al. [78] the genetic diversity of the olive germplasm of the oasis of Degache, in the south west part of Tunisia, characterized by arid climate and low pluviometry (<100 mm/year), showing the presence of olive genotypes largely diverse from the reference germplasm, including the traditional cultivar of Chetoui and Chemlali, but also from other modern varieties [78]. The population structure analysis identified two gene pools more represented in germplasm from southern Tunisia, where environmental conditions at critical plant development phases, are harsher. This suggests that this germplasm might present traits of adaptation which could be useful for breeding to improve resilience to abiotic stresses.

## 4. Future Perspectives

Collection, characterization, conservation and evaluation of genetic resources are crucial steps to preserve them from genetic erosion and to offer plant material available for future use in breeding programs [26].

The development of technologies for next generation sequencing (NGS) is producing a high amount of data, offering the opportunity to explore the relationships between genetic and phenotypic diversity in the olive tree. In addition, the recent advances in omics (transcriptomics, proteomics, metabolomics) are providing new tools for a better understanding of the molecular mechanism involved in the development of traits of interest, thereby facilitating their use in selection programs.

Alongside molecular characterization, high-throughput phenotyping platforms are being developed, which make it possible to rapidly phenotype a large number of plants at a reduced cost and time compared to traditional techniques, therefore accelerating the timing of breeding [80].

In the last twenty years, olive breeding has been focused on the productivity and quality of the oil, but following the climate changes taking place worldwide, the attention is now turning rather towards greater tolerance of the trees to abiotic stresses, in particular drought. More than 90% of Tunisian olive trees are non-irrigated, leaving Tunisia particularly vulnerable. This has led to the initiation of olive breeding programs focusing on the use of local olive germplasm and intraspecific crossbreeding of cultivars of well-known value to combine their advantageous qualities [81]. Thus, we look with increasing interest at the natural sources of useful alleles, which is the traditional germplasm that had been neglected in the past years. Locally adapted cultivated varieties (“landraces” or “farmers’ varieties”) and wild relatives of crops are rich sources of genetic diversity, and they can provide important keys in helping to build resilience in agriculture. Their conservation in their natural habitats in the case of wild species, or in the locations where they are cultivated in the case of landraces/farmers’ varieties, is essential to maintaining this diversity which is continually adapting to local environmental conditions.

By combining molecular with phenotypic data in meta-analyses and applying new approaches such as Quantitative Trait Loci mapping, genome-wide association studies, and genomic selection, it will be possible to explore the potential of cultivars and ecotypes statically stored in gene banks and other collections, accelerating olive breeding programs for achieving the desired improvements in olive cultivars.

However, *in situ* and *ex situ* conservation are currently quite unplanned and uncoordinated, and to streamline and strengthen our efforts, we need effective and permanent support mechanisms that are in place for optimal utilization. Research is needed to identify gaps in existing *ex situ* collections in order to ensure that the entire gene pool is adequately represented, for broadening the exploration of crop diversity, and reducing redundancy by eliminating duplicates. At the same time, it is necessary to act at management level, to ensure accessibility to all information and facilitate their use through a further development of specific subsets of varieties that can be actively used in breeding programs for a genetic enhancement and broadening the genetic base aims.

Finally, it will be important a further effort to promoting the use and commercialization of marginal varieties, by enlarging networks and information systems, and by strengthening the public awareness about the importance of plant genetic resources in developing a sustainable agriculture. 

## Figures and Tables

**Table 1:** List of the 9 microsatellite markers (SSR) used for routine genotyping of olive accessions. For each SSR, the identification code (SSR ID), bibliographic reference, repeat motif, primer sequence and annealing temperature (Ta) are reported.

SSR ID	Bibliographic Reference	Repeat Motif	Primer Sequence (5′-3′)	Ta
DCA03	Sefc et al. (2000) [21]	(GA)_19_	cccaagcggaggtgtatattgttac	50 °C
			tgcttttgtgtttgagatgttg	
DCA05	Sefc et al. (2000) [21]	(GA)_15_	aacaaatcccatacgaactgcc	50 °C
			cgtgttgctgtgaagaaaatcg	
DCA09	Sefc et al. (2000) [21]	(GA)_23_	aatcaaagtcttccttctcatttcg	55 °C
			gatccttccaaaagtataacctctc	
DCA15	Sefc et al. (2000) [21]	(CA)_3_G(AC)_14_	gatcttgtctgtatatccacac	50 °C
			tataccttttccatcttgacgc	
DCA16	Sefc et al. (2000) [21]	(GT)_13_(GA)_29_	ttaggtgggattctgtagatggttg	50 °C
			ttttaggtgagttcatagaattagc	
DCA17	Sefc et al. (2000) [21]	(GT)_9_(AT)_7_AGATA(GA)_38_	gatcaaattctaccaaaaatata	50 °C
			taatttttggcacgtagtattgg	
DCA18	Sefc et al. (2000) [21]	(CA)_4_CT(CA)_3_(GA)_19_	aagaaagaaaaaggcagaattaagc	50 °C
			gttttcgtctctctacataagtgac	
EMOL	De la Rosa et al. (2002) [34]	(GA)_12_	ctttccaatatgggctctcg	55 °C
			atggcactttacgggaaaaa	
			tgccaattatggggctaact	
GAPU101	Carriero et al. (2002) [22]	(GA)_8_(G)_3_(AG)_3_	catgaaaggagggggacata	57–60 °C
			ggcacttgttgtgcagattg	
GAPU71b	Carriero et al. (2002) [22]	GA(AG)_6_(AAG)_8_	gatcaaaggaagaaggggataaa	57–60 °C
			acaacaaatccgtacgcttg	
UDO28	Cipriani et al. (2002) [22]	(CA)_23_(TA)_3_	ctgcagcttctgcccatac	57 °C
			gcagatcatcatttggcact	
UDO43	Cipriani et al. (2002) [22]	(GT)_12_	tcggctttacaacccatttc	57 °C
			tgccaattatggggctaact	

**Table 2:** List of accessions conserved at Tunisian olive national collection of Boughrara, accessible at www.iosfax.agrinet.tn.

N°	Accession Name	N°	Accession Name
1	BAROUNI	28	JEDDARIA CHAAL
2	BELDI	29	JEMRI_BOUCHOUKA
3	BESBESSI	30	LATTOUT SNED
4	BIDH_HMAM	31	LQAM EL KOTTI
5	CHAHLEYA	32	MALLAHI EL MOUAMMAR
6	CHEMCHALI_GAFSA	33	MARSALINE
7	CHEMLALI BALHI	34	MARSALINE
8	CHEMLALI BOUCHOUKA	35	MBAZZEL KBIR
9	CHEMLALI CHOUAMEKH	36	MESKI
10	CHEMLALI LACH4HAB	37	NEB TATAOUINE
11	CHEMLALI ONTHA TATAOUINE	38	NEB_JEMAL_TATAOUINE
12	CHEMLALI SIG	39	OUESLATI2
13	CHEMLALI_JERBA	40	RKHAMI3
14	CHEMLALI_ONTHA	41	SAYALI
15	CHEMLALI_SFAX	42	SEMNI JBENIANA
16	CHEMLALI_TATAOUINE	43	TOFFAHI
17	CHEMLALI_TATAOUINE	44	TOUNSI
18	CHEMLALI_ZARZIS	45	TOUNSI GAFSA
19	CHETOUI2	46	ZALMATI
20	DHOKKAR NAFTI	47	ZARBOUT LOUZIR
21	ECH CHAHLA	48	ZARRAZI EJJBAL
22	FAKHARI	49	ZARRAZI KGH
23	FAKHARI TATAOUINE	50	ZARRAZI_ZARZIS
24	GERBOUI2	51	ZEITOUN BOUBAZZOULA
25	HORR CHARQIA	52	ZEITOUN EL MANACHER
26	HORR EL KOTTI	53	ZEITOUN KHDIM EL BEY
27	INJASSI HCHICHINA	54	ZEYETI EL KOTTI

## Data Availability

Data available in www.iosfax.agrinet.tn (accessed on 3 February 2021).
